# Ten Simple Rules to Enable Multi-site Collaborations through Data Sharing

**DOI:** 10.1371/journal.pcbi.1005278

**Published:** 2017-01-19

**Authors:** Mary Regina Boland, Konrad J. Karczewski, Nicholas P. Tatonetti

**Affiliations:** 1 Department of Biomedical Informatics, Columbia University, New York, New York, United States of America; 2 Department of Systems Biology, Columbia University, New York, New York, United States of America; 3 Department of Medicine, Columbia University, New York, New York, United States of America; 4 Observational Health Data Sciences and Informatics, Columbia University, New York, New York, United States of America; 5 Program in Medical and Population Genetics, Broad Institute of MIT and Harvard, Cambridge, Massachusetts, United States of America; 6 Analytic and Translational Genetics Unit, Massachusetts General Hospital, Boston, Massachusetts, United States of America

Open access, open data, and software are critical for advancing science and enabling collaboration across multiple institutions and throughout the world. Despite near universal recognition of its importance, major barriers still exist to sharing raw data, software, and research products throughout the scientific community. Many of these barriers vary by specialty [1], increasing the difficulties for interdisciplinary and/or translational researchers to engage in collaborative research. Multi-site collaborations are vital for increasing both the impact and the generalizability of research results. However, they often present unique data sharing challenges. We discuss enabling multi-site collaborations through enhanced data sharing in this set of Ten Simple Rules.

Collaboration is an essential component of research [2] that takes many forms, including internal (across departments within a single institution) and external collaborations (across institutions). However, multi-site collaborations with more than two institutions encounter more complex challenges because of institutional-specific restrictions and guidelines [3]. Vicens and Bourne focus on collaborators working together on a shared research grant [4]. They do not discuss the specific complexities of multi-site collaborations and the vital need for enhanced data sharing in the multi-site and large-scale collaboration context, in which participants may or may not have the same funding source and/or research grant.

While challenging, multi-site collaborations are equally rewarding and result in increased research productivity [5, 6]. One highly successful multi-site and translational collaboration is the Electronic Medical Records and Genomics (eMERGE) network (URL: https://emerge.mc.vanderbilt.edu/) initiated in 2007 [7]. The eMERGE network links biorepository data with clinical information from Electronic Health Records (EHRs). They were able to find novel associations and replicate many known associations between genetic variants and clinical phenotypes that would have been more difficult without the collaboration [8]. eMERGE members also collaborated with other consortiums and networks, including the Alzheimer’s Disease Genetics Consortium [9] and the NINDS Stroke Genetics Network [10], to name a few. Other successful collaborations include OHDSI: Observational Health Data Sciences and Informatics (http://www.ohdsi.org/), which builds off of the methodology from the Observational Medical Outcomes Partnership (OMOP) [11], and CIRCLE: Clinical Informatics Research Collaborative (http://circleinformatics.org/). In genetics, there are many consortiums, including ExAC: The Exome Aggregation Consortium (http://exac.broadinstitute.org/), the 1000 Genomes Project Consortium (http://www.1000genomes.org/), the Australian BioGRID (https://www.biogrid.org.au/), The Cancer Genome Atlas (TCGA) (http://cancergenome.nih.gov/), Genotype-Tissue Expression Portal (GTEx: http://www.gtexportal.org/home/), and Encyclopedia of DNA Elements at UCSC (ENCODE: https://genome.ucsc.edu/ENCODE/) among others.

Based on our experiences as both users and participants in collaborations, we present ten simple rules on how to enable multi-site collaborations within the scientific community through enhanced data sharing. The rules focus on understanding privacy constraints, utilizing proper platforms to facilitate data sharing, thinking in global terms, and encouraging researcher engagement through incentives. We present these ten rules in the form of a pictograph of modern life (**Fig 1**), and we provide a table of example sources and sites that can be referred to for each of the ten rules (**Table 1**). Please note that this table is not meant to be exhaustive, only to provide some sample resources of use to the research community.

**Fig 1:**
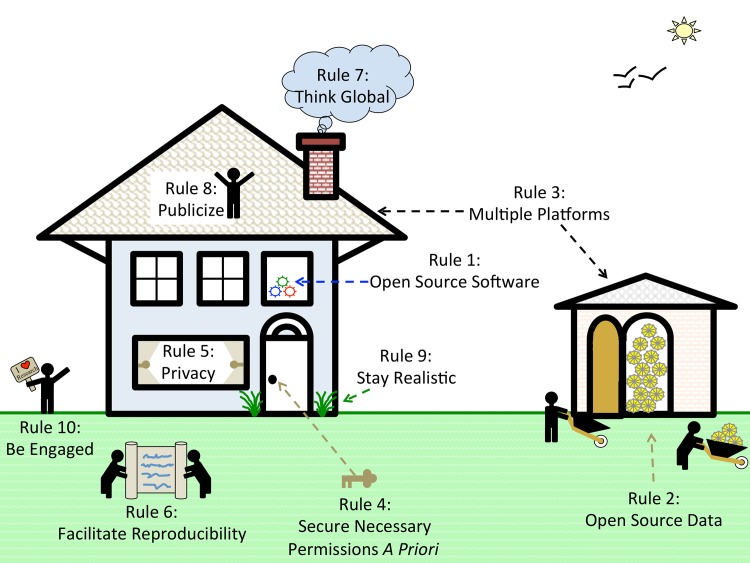
Modern life context for the ten simple rules. This figure provides a framework for understanding how the “Ten Simple Rules to Enable Multi-site Collaborations through Data Sharing” can be translated into easily understood modern life concepts. **Rule 1** is Open-Source Software. The openness is signified by a window to a room filled with algorithms that are represented by gears. **Rule 2** involves making the source data available whenever possible. Source data can be very useful for researchers. However, data are often housed in institutions and are not publicly accessible. These files are often stored externally; therefore, we depict this as a shed or storehouse of data, which, if possible, should be provided to research collaborators. **Rule 3** is to “use multiple platforms to share research products.” This increases the chances that other researchers will find and be able to utilize your research product—this is represented by multiple locations (i.e., shed and house). **Rule 4** involves the need to secure all necessary permissions a priori. Many datasets have data use agreements that restrict usage. These restrictions can sometimes prevent researchers from performing certain types of analyses or publishing in certain journals (e.g., journals that require all data to be openly accessible); therefore, we represent this rule as a key that can lock or unlock the door of your research. **Rule 5** discusses the privacy issues that surround source data. Researchers need to understand what they can and cannot do (i.e., the privacy rules) with their data. Privacy often requires allowing certain users to have access to sections of data while restricting access to other sections of data. Researchers need to understand what can and cannot be revealed about their data (i.e., when to open and close the curtains). **Rule 6** is to facilitate reproducibility whenever possible. Since communication is the forte of reproducibility, we depicted it as two researchers sharing a giant scroll, because data documentation is required and is often substantial. **Rule 7** is to “think global.” We conceptualize this as a cloud. This cloud allows the research property (i.e., the house and shed) to be accessed across large distances. **Rule 8** is to publicize your work. Think of it as “shouting from the rooftops.” Publicizing is critical for enabling other researchers to access your research product. **Rule 9** is to “stay realistic.” It is important for researchers to “stay grounded” and resist the urge to overstate the claims made by their research. **Rule 10** is to be engaged, and this is depicted as a person waving an “I heart research” sign. It is vitally important to stay engaged and enthusiastic about one’s research. This enables you to draw others to care about your research.

**Table 1:** Example sources and sites for each of the ten simple rules.

Rule	Example	Site
**Rule 1: Make Software Open-Source**		
	Github	https://github.com
	CRAN	https://cran.r-project.org
	Bioconductor	https://www.bioconductor.org
**Rule 2: Provide Open-Source Data** (When Possible)		
*Deposit Source Data in Appropriate Repositories*		
	Sequence Read Archive (SRA)	https://www.ncbi.nlm.nih.gov/sra
	Gene Expression Omnibus (GEO)	https://www.ncbi.nlm.nih.gov/geo
	ClinVar	https://www.ncbi.nlm.nih.gov/clinvar
*Consider Middle-Ground Data Sharing Approaches for Sensitive Data*		
	dbGaP	https://www.ncbi.nlm.nih.gov/gap
	Shared Health Research Information Network (SHRINE)	https://catalyst.harvard.edu/services/shrine
	BioGrid Australia	https://www.biogrid.org.au
**Rule 3: Use Multiple Platforms to Share Research Products**		
	Figshare	https://figshare.com
	Github	https://github.com
	ExAC Browser	http://exac.broadinstitute.org
	Google Forums	
**Rule 4: Secure Necessary Permissions/Data Use Agreements A Priori**		
*Guides for Creating a DUA*		
	Department of Health and Human Services Best Practice Guide for DUA	http://www.hhs.gov/ocio/eplc/EPLC%20Archive%20Documents/55-Data%20Use%20Agreement%20(DUA)/eplc_dua_practices_guide.pdf
	Health Care Systems Research Network DUA Toolkit	http://www.hcsrn.org/en/Tools%20&%20Materials/GrantsContracting/HCSRN_DUAToolkit.pdf
*Example DUAs*		
	NASA DUA	http://above.nasa.gov/Documents/NGA_Data_Access_Agreement_new.pdf
	SEER-MEDICARE DUA	https://healthcaredelivery.cancer.gov/seermedicare/obtain/seerdua.docx
**Rule 5: Know the Privacy Rules for Your Data**		
	Health Insurance Portability and Accountability Act (HIPAA)	http://www.hhs.gov/hipaa/for-professionals/privacy
**Rule 6: Facilitate Reproducibility**		
*Resources for Increasing Research Reproducibility*		
	MetaSub Research Integrity and Reproducibility	http://metasub.org/research-integrity-and-reproducibility/
	Reproducibility and Open Science Working Group—GitHub	http://uwescience.github.io/reproducible/guidelines.html https://github.com/uwescience/reproducible
*Example Projects with Assessed Reproducibility*		
	eMERGE PheKB	https://phekb.org/network-associations/emerge
**Rule 7: Think Global**		
*Guides for Collaborating Globally*		
	National Academies “Collaborating with Foreign Partners to Meet Global Challenges”Resources	http://sites.nationalacademies.org/PGA/PGA_041691
	Global Alliance for Genomics and Health	http://genomicsandhealth.org/work-products-demonstration-projects/catalogue-global-activities-international-genomic-data-initiati
	The Global Strategy of the US Department of Health and Human Services	http://www.hhs.gov/sites/default/files/hhs-global-strategy.pdf
*Examples of Successful International Projects*		
	Human Fertility Database	http://www.humanfertility.org/cgi-bin/main.php
	Human Mortality Database	http://www.mortality.org
**Rule 8: Publicize Your Work**		
*Research Without Novelty Requirement*		
	*PLOS ONE*	http://journals.plos.org/plosone
	*Scientific Reports*	http://www.nature.com/srep
	*Cell Reports*	http://www.cell.com/cell-reports/home
*Data Resources (Web Browsers*, *Databases)*		
	*Scientific Data*	http://www.nature.com/sdata
	*Database*	https://database.oxfordjournals.org
*Pure Open Science Research (all data must be open)*		
	F1000	https://f1000research.com
**Rule 9: Stay Realistic**		
	Retraction Watch	retractionwatch.com
**Rule 10: Be Engaged**		
*Resources to Facilitate Researcher Engagement*		
	KNAER Creating Partnerships: Learning New Ways to Connect	http://www.knaer-recrae.ca/blog-news-events
*Example Projects with Researcher Engagement*		
	STAN	http://mc-stan.org
	STAN “swag”	http://mc-stan.org/shop

## Definitions

In this paper, we use the term “research product” to include all results from research. This includes algorithms, developed software tools, databases, raw source data, cleaned data, and various metadata generated as a result of the research activity. We differentiate this from “data,” which comprises the primary “facts and statistics collected together for analysis” for that particular collaboration. Therefore, data could include genetic data or clinical data. By these definitions, developed software tools are not “data” but “research products.” Novel genetic sequences collected for analysis would be considered “raw source data,” which is a type of “research product.”

## Rule 1: Make Software Open-Source

The cornerstone of facilitating multi-site collaborations is to enhance data sharing and make software open-source [12]. By allowing the source code to be open, researchers allow others to both reproduce their work and build upon it in novel ways. To engage in multi-site collaborations, it is necessary for collaborators to have access to code in a repository that is shared among collaborators (although, this could be private and not open to the general public). When the study is complete and the paper is under review and/or published, a stable copy of the code should be made available to the general public. Internal sharing allows the code to be developed, while public sharing of a stable version allows the code to be refined and built upon by others.

Many researchers still limit access to their work despite the known advantages of making software open-source upon publication (e.g., higher impact publications [5]). For example, they allow users to interact with their algorithm by inputting data and receiving results on a web platform, while the backend algorithm often remains inaccessible. Masum et al. advocate the reuse of existing code in their Ten Simple Rules for cultivating open science [13]. However, this is often easier said than done. As long as the backend algorithms remain hidden, open science will not be possible. Therefore, it is essential for researchers interested in participating in multi-site collaborations to make their software code and algorithms open. Because making software truly “open” can be complex, Prlic and Proctor provide Ten Simple Rules to assist researchers in making their software open-source [12]. Truly open-source software is an essential component in collaborations [13]. Openness also has advantages for the researchers themselves. With more eyes on the source code, others within the community can refine the code, leading to greater identification and correction of errors. There are several methods for sharing software code. If you use the R platform, then libraries can be shared with the entire open-source community via CRAN (https://cran.r-project.org/) and bioconductor, which is specifically for biologically related algorithms (https://www.bioconductor.org/). Code can also be shared on Github with issue trackers for error detection.

## Rule 2: Provide Open-Source Data

### Deposit Source Data in Appropriate Repositories

Whenever possible, it is important to make source data available. Openness benefits your collaborators by allowing them to perform additional analyses easily. Source data could include not only processed or cleaned data used in algorithms but also raw data files. These files can often be very large; therefore, they are often stored in some external site or data warehouse. The National Center for Biotechnology Information (NCBI) maintains the Sequence Read Archive (SRA) (https://www.ncbi.nlm.nih.gov/sra) and the Gene Expression Omnibus (GEO) (https://www.ncbi.nlm.nih.gov/geo/); both are great places to deposit source data, if appropriate.

In addition to raw data files, it is also helpful to provide intermediate data files at various stages of processing. If comparing your results to those in the literature, it can also be useful to provide a meta-analysis with publications (along with PubMed IDs) that detail those publications that support and refute the results you obtained.

Data sharing is vitally important for multi-site collaborations by allowing researchers to compare results from across vastly different study populations, which increases the generalizability of the findings [14]. While a multi-site research project is still ongoing, data can be shared in a private shared space until all necessary data quality checks have been conducted and the findings have been published. After publication, data can be deposited in GEO, SRA, ClinVar (https://www.ncbi.nlm.nih.gov/clinvar/), and any other domain-specific sites that are appropriate for source data deposition.

### Consider Middle-Ground Data Sharing Approaches for Sensitive Data

Raw source data is not always fully shareable with the public. This can be because of data use restrictions (see rule 4) or privacy concerns (see rule 5). Alternative mechanisms exist for sharing portions of data with the research community. For example, the database for Genotypes and Phenotypes or dbGaP (https://www.ncbi.nlm.nih.gov/gap) provides data holders with two levels of access: open and controlled. The open selection allows for broad release of nonsensitive data online, whereas the controlled release allows sensitive datasets to be shared with other investigators, provided certain restrictions are met. This increases the ability for researchers to share portions of their data that would not be shareable otherwise.

In addition to the restricted data sharing option provided by dbGaP, others have looked at ways of developing middle-ground approaches for sharing sensitive raw data or metadata. Several of these mid-level approaches use Federated Access systems that allow researchers to query databases containing sensitive data while preventing direct access to the data itself. An example within the United States is the Shared Health Research Information Network (SHRINE), which provides a Federated system that is HIPAA compliant [15]. International groups have also seen success in this area. BioGrid Australia (https://www.biogrid.org.au/) allows researchers to access hundreds of thousands of health records through a linked data platform where individual data holders maintain control of their data [16]. Researchers can then be provided with authorized access to certain elements within the data while restricting access to private sections of the medical data. These mid-level approaches facilitate collaboration both within the institution (i.e., across departments) and across institutions by allowing researchers to access sensitive data indirectly. They can even match patients to similar patients (for association analyses) while maintaining stringent privacy constraints [17]. Others provide summary statistics computed over large cohorts (e.g., ExAC browser/database), which maintains privacy while providing others with important information about the populations that can be used in subsequent analyses and comparisons.

## Rule 3: Use Multiple Platforms to Share Research Products

To collaborate with researchers from different backgrounds, it is often necessary to use multiple platforms when sharing data (as different disciplines often have different policies). Using multiple platforms allows individuals from diverse backgrounds to have access to your research product. General phrases like “open data” and “open science” are phrases used commonly in the research community but provide little direction [13]. Research products take many different forms, including 1) raw source data regardless of collection type (e.g., health data, genomic data, survey data, and epidemiological data), 2) software code (mentioned in rule 1), and 3) metadata elements and results of computations used to generate figures published in scientific research. Some data types cannot be fully shared (e.g., EHR data—see rule 5), but most algorithms and summary results/statistics are shareable.

Each of these types of open data necessitates a different platform for data sharing. Figshare (https://figshare.com/) allows users to share data involving published figures. Github (https://github.com/) allows users to share code that is in development or published. For code that is well developed, open-source packages can be created, for example, an R library, which can be deposited in CRAN or bioconductor. R libraries can be shared immediately on github without any code checking—this is advisable for code that is still in development. However, when code is finalized, it can be submitted to bioconductor as an R library. Approved libraries are vetted to ensure that the code works well. Vignettes are also good to write to help new users get used to the R package. When collaborating across multiple sites, it is also important to have vignettes and sample source data to help users learn how to use the code even if R is not your language of choice. Data formats, differences among formats, and programming languages are important to consider when sharing data across multiple platforms. Different platforms often have different required formats. While it may seem tedious to translate code, source data, and documentation across multiple formats and data schemas, it can be very helpful, and it will increase the number of users that will find your data and results interesting.

To facilitate communication among members of a collaborative effort, there are many options, including Google forums and wiki webpages, among others. Others have specially designed websites for the sole purpose of allowing users to browse and download the data directly; one such website is the ExAC Browser (http://exac.broadinstitute.org/), which integrates data obtained from 17 different consortiums (http://exac.broadinstitute.org/about) [18].

## Rule 4: Secure Necessary Permissions/Data Use Agreements A Priori

Some datasets have provisos that affect publication, and these need to be addressed a priori. For example, the ability for researchers to publish an algorithm that uses a Government dataset can depend on the department that generated the data. For example, certain National Aeronautics and Space Administration (NASA) datasets stipulate that data usage requires users to add certain NASA employees to subsequent publications. This is an important stipulation. Others may disallow the deposition of data into an “open” platform as part of their data use agreements (http://above.nasa.gov/Documents/NGA_Data_Access_Agreement_new.pdf). These stipulations can hinder researchers attempting to produce transparent science.

Other datasets have data use agreements as an added layer to ensure that patients are protected. For example, the Surveillance, Epidemiology, and End Results (SEER) dataset linked with Medicare (i.e., SEER-Medicare dataset) requires that users submit the intended publication to their offices for pre-submission approval. This can seem burdensome to researchers; however it is a condition of the data use agreement and, therefore, must be complied with. Researchers need to be aware of all provisos when including such data in their studies. Before publishing, or providing data in any type of platform whether open, restricted, or closed, it is important to secure all necessary provisions and data use agreements.

## Rule 5: Know the Privacy Rules for Your Data

Data come with many caveats. For this reason, it is important to understand what you can and cannot do (i.e., the privacy rules) with your data. Keeping and maintaining data privacy is different from data use agreements (DUA, see rule 4). For example, data that is not sensitive may have restrictive DUAs for other reasons (e.g., data from a collaborator in industry). Also, privacy rules often involve your own source data, whereas DUAs become necessary when using data from collaborators or a government source.

Certain datasets, e.g., genomic and EHR data, may be impossible to fully publish on an open platform due to the Health Insurance Portability and Accountability Act (HIPAA) privacy rules and other privacy concerns related to patient re-identifiability (http://www.hhs.gov/hipaa/for-professionals/privacy/). Therefore, it is important to know the privacy stipulations of **all** data used in your collaborations and how this affects the ability to share results among members of the team (especially when members of the team are at different institutions). Methods that anonymize patient information while allowing patient-level data sharing may be the way of the future [19]. However, institutional-specific policies and/or country-specific laws can limit or prevent usage of such methods. This is an important item to consider and discuss with all collaborators at the outset of any collaboration. We discuss some methods that can be used to provide some forms of sensitive data in a shareable federated space in rule 2.

## Rule 6: Facilitate Reproducibility

Another aspect of both data sharing and enabling multi-site collaborations is reproducibility. Sandve et al. provide Ten Simple Rules for facilitating research reproducibility in general [20]. Keeping track of research results and how data were generated is vital for reproducibility [20]. This site-level record keeping becomes vital when engaging in multi-site collaborations. If one aspect of a methodology is not conducted in the same way at one site, the overall results can be affected in drastic ways. In other words, reproducibility is a core requirement for successful collaborations.

In genetics and computational biology, the issue of standardizing results from across different types of gene sequencing platforms is a major issue [21]. Researchers that use a mixture of clinical and genetic data (for Phenome-Wide Association Studies, PheWAS [22]) often depend on local EHR terminology systems for identifying patient populations. Therefore, standard phenotype definitions are required and must be harmonized across multiple sites to ensure that the definitions are accurate at each site [23]. Several multi-site collaborations have developed platforms that provide links to all necessary documentation, code, and data schemas to help facilitate this process [24], including the eMERGE network. This step is integral to data sharing and enabling multi-site collaborations.

## Rule 7: Think Global

The importance of thinking globally cannot be overstated. Health care, genetics, climate, and all aspects of science affect the world as a whole. Therefore, it is important to think globally when performing scientific research. Most software languages are designed to be agnostic to the local language of the country. However, understanding and using these languages requires adequate documentation and user manuals to be provided in the local languages of the programmers/implementers. Despite this, open-source languages often provide user manuals in certain languages. For example, R is a popular open-source language yet has official documented translations in only four languages: English, Russian, German, and Chinese (https://www.r-project.org/other-docs.html). Problems can surface when collaborators in different regions run into difficulties with running R. This affects data sharing on a global scale and should be considered when collaborating on an international venue.

Translational mechanisms may also be necessary to understand and to harmonize country-specific terminology. This is especially important as definitions for obesity and many psychiatric conditions vary widely across the globe [25]. Even seemingly simple biological features (e.g., tall versus short) can be difficult to translate in global terms. For example, an average height Norwegian may appear to be tall in a different country. Translating biological features to common absolute metrics (e.g., height) helps to alleviate ambiguities that can occur from categorical variables. Certain diseases, especially psychiatric conditions, are extremely important to study at the multi-site level to increase the generalizability of the results [14]. However, psychiatric conditions are more difficult to translate without a thorough knowledge of how the condition is defined in the underlying country or region [25]. Solutions often involve using concrete measures, e.g., brain imaging analysis, versus subjective measures such as depression presence or absence [14].

There are many layers to thinking on a global scale. There are mechanical differences (i.e., the software language and documentation) and also the conceptual differences (i.e., country- or region-specific medical definitions). Organizations such as the World Health Organization work tirelessly to integrate different conceptual interpretations of diseases into a standard guideline. Using these guidelines and not a country-specific guideline helps your research work reach the broader scientific community.

Several groups have successfully integrated data across multiple countries and provided their data in an open form. The Max Planck Institute for Demographic Research (MPIDR) in Germany collaborated with two separate groups to produce two databases containing international data. Both datasets contain integrated results from over 30 countries. Additionally, all finished data (after cleaning) is made available to users in an open format via two specially designed databases: the Human Fertility Database (http://www.humanfertility.org/cgi-bin/main.php) [26] and the Human Mortality Database (http://www.mortality.org/) [27]. Only cleaned data are returned to users in a standardized format, allowing users to easily compare countries with one another. The MPIDR collaborated with the Vienna Institute of Demography (Austria) in creating the Human Fertility Database and the University of California, Berkeley for the Human Mortality Database. They provide a good example of a group that successfully harmonized definitions across countries by overcoming international barriers, and they provided data back to researchers in an easily useable and standardized format. The group provides detailed descriptions of how they harmonized various timescales across countries in a methods document (http://www.humanfertility.org/Docs/methods.pdf) that could easily be submitted as a research report (see Rule 6).

## Rule 8: Publicize Your Work

Publishing all aspects of your work in the appropriate venues is vital for maintaining a multi-site collaboration. This enables each aspect of your research to be assessed by appropriate peer reviewers. Publishing different aspects of your work in separate papers in separate journals allows your contributions to be seen by those most able to learn from your work. Remember, it is important to make your research work available to those who can benefit from your results. Depending on your findings, this can include methodologists, clinicians, epidemiologists, geneticists, and others.

New journals have been developed recently to facilitate open science, which are focused on certain aspects of research. For instance, there are several journals that do not require novelty as a requirement such as *PLOS ONE*, *Scientific Reports*, and *Cell Reports*. These journals are good choices for research results that may be part of a larger research project or collaborative but are not inherently novel. Other journals, such as *Scientific Data* and *Database*, are good choices for publishing a resource containing your collected research source data. It is often advisable to publish in data-focused journals simultaneously with an algorithm or results-focused paper that highlights the novel aspects of your research. In some cases, data can be published afterwards if it is part of a large collaborative and the database or user-interface is in production at the time that the main contribution is published.

Publishing in multiple venues is highly important for those engaged in multi-site collaborations, because these projects often involve a tremendous investment of time and resources from across many different organizations. Therefore, it is vital to highlight each and every research contribution that the collaboration has generated to facilitate further engagement from the community. If you are able to provide all raw source data on an open platform, there are new journals designed specifically to facilitate open science such as F1000 (https://f1000research.com/) that may be worth considering. F1000 is also a great source for intermediate results such as posters, which collaborators may have presented at various conferences while working towards the final finished paper. After publication, some collaborative groups effectively utilize blogging (both macro and micro) to communicate with other researchers and the general public. However, it is also important not to overstate the claims in any paper submission/publication or media regarding that publication but to stay focused on the individual contribution of that particular work.

## Rule 9: Stay Realistic, but Aim High

When performing quality research, and collaborating with others, it is important not to overstate the claims of your research—either in publication or online. It is vitally important to resist the urge to overstate the claims and to remain both humble and grounded. This is critical in collaborations because if a researcher overstates the claim in a paper, or worse, shares data publicly that he or she is unable to do legally (e.g., via the stipulations in a DUA), then the paper may be retracted. This could result in irreparable damage to the collaborative group.

This rule also links back to rule 2—making the source data available. This allows others in the research community to check your work interactively, which can help prevent overstating research claims [28]. A site exists that posts retracted journal articles on a public forum, retractionwatch.com. The site includes not only instances of plagiarism and fabrication of data but also papers that are retracted due to human error on the part of an experiment (e.g., a protocol was not followed exactly as specified in the paper) or on the part of the analysis (e.g., the wrong type of statistical test was performed, making the conclusions not substantiated by the data).

So, stay realistic, but do not be afraid to challenge the status quo. Some of the most respected research today was research that challenged the current understanding of the leading scientists at that point in time; this includes the seminal works on Pangaea and even that DNA is composed of a double helix. These concepts were earth-shattering at the time and could have been completely wrong, but the researchers backing them were not afraid to make their theories, data, and results public. These are the things that change science. So, remain humble, do not intentionally overstate the claims of your research, but at the same time do not be afraid to challenge the current mindset and way of thinking. You may be completely off, or you may just be a groundbreaking innovator.

## Rule 10: Be Engaged

Be engaged with those using your research, your data, and your code. Communicate with them using various software social platforms—Github, figshare, and so forth. Respond readily when users have questions and concerns. Attempt to follow the motto—release early, release often. Engage with researchers in non-traditional ways. For example, several collaborative efforts have created their own gear, e.g., t-shirts, to engage the community. One such collaborative is the open-source statistical modeling language—STAN (http://mc-stan.org/). They have created their own line of STAN “swag” (http://mc-stan.org/shop/) to facilitate user engagement. Communicate often with the research community to convince them your research is worth caring about. The bottom line in collaboration is to care deeply about your research. If you care and you make it known that you care deeply about the problem, then it becomes possible to convince others that your research is important.

## Concluding Remarks

Collaborations, especially large, multi-site collaborations, contain a lot of pitfalls that must be overcome. In this paper, we present ten simple rules that will help researchers share their data and methods to facilitate successful and meaningful multi-site collaborations. We describe these rules and highlight several successful multi-site collaborations.
